# Gene correction of *HBB* mutations in CD34^+^ hematopoietic stem cells using *Cas9* mRNA and ssODN donors

**DOI:** 10.1186/s40348-018-0086-1

**Published:** 2018-11-14

**Authors:** Justin S. Antony, Ngadhnjim Latifi, A. K. M. Ashiqul Haque, Andrés Lamsfus-Calle, Alberto Daniel-Moreno, Sebastian Graeter, Praveen Baskaran, Petra Weinmann, Markus Mezger, Rupert Handgretinger, Michael S. D. Kormann

**Affiliations:** 1 Department of Pediatrics I, Pediatric Infectiology and Immunology, Translational Genomics and Gene Therapy in Pediatrics, University of Tuebingen, Tuebingen, Germany; 2 University Children’s Hospital, Department of Pediatrics I, University of Tuebingen, Tuebingen, Germany; 3 Department of Hematology, Oncology, Clinical Immunology, University of Tuebingen, Tuebingen, Germany; 4 Center for Quantitative Biology, University of Tuebingen, Tuebingen, Germany

**Keywords:** *HBB*, Beta-thalassemia, Gene correction, CRISPR/Cas9, IVS1–110, *Cas9* mRNA

## Abstract

**Background:**

β-Thalassemia is an inherited hematological disorder caused by mutations in the human hemoglobin beta (*HBB*) gene that reduce or abrogate β-globin expression. Although lentiviral-mediated expression of β-globin and autologous transplantation is a promising therapeutic approach, the risk of insertional mutagenesis or low transgene expression is apparent. However, targeted gene correction of *HBB* mutations with programmable nucleases such as CRISPR/Cas9, TALENs, and ZFNs with non-viral repair templates ensures a higher safety profile and endogenous expression control.

**Methods:**

We have compared three different gene-editing tools (CRISPR/Cas9, TALENs, and ZFNs) for their targeting efficiency of the *HBB* gene locus. As a proof of concept, we studied the personalized gene-correction therapy for a common β-thalassemia splicing variant *HBB*^IVS1–110^ using *Cas*9 mRNA and several optimally designed single-stranded oligonucleotide (ssODN) donors in K562 and CD34^+^ hematopoietic stem cells (HSCs).

**Results:**

Our results exhibited that indel frequency of CRISPR/Cas9 was superior to TALENs and ZFNs (*P* < 0.0001). Our designed sgRNA targeting the site of *HBB*^IVS1–110^ mutation showed indels in both K562 cells (up to 77%) and CD34^+^ hematopoietic stem cells—HSCs (up to 87%). The absolute quantification by next-generation sequencing showed that up to 8% site-specific insertion of the *Nhe*I tag was achieved using *Cas9* mRNA and a chemically modified ssODN in CD34^+^ HSCs.

**Conclusion:**

Our approach provides guidance on non-viral gene correction in CD34^+^ HSCs using *Cas*9 mRNA and chemically modified ssODN. However, further optimization is needed to increase the homology directed repair (HDR) to attain a real clinical benefit for β-thalassemia.

**Electronic supplementary material:**

The online version of this article (10.1186/s40348-018-0086-1) contains supplementary material, which is available to authorized users.

## Background

β-Thalassemia (OMIM: 613985) is an inherited hematological disorder caused by mutations of the human hemoglobin beta (*HBB*) gene, leading to deficient β-globin expression and severe anemia [1]. The current treatment options include allogeneic bone marrow transplantation and hematopoietic stem cell (HSC) transplantation but are limited to histo-compatible donors. However, gene therapy based on autologous transplantation of a lentiviral-transferred *HBB* gene to HSCs resulted in remarkable clinical benefit [2, 3]. Though the safety and efficacy of lentiviral-based gene therapy is positive in a treated patient, the transactivation of the proto-oncogene *HMGA2* and more than 3500 unique integration sites in tested mouse model forewarns the possibility of insertional mutagenesis [2, 4]. Earlier retroviral gene therapy studies on other inherited diseases reported the treatment-related leukemogenesis [5–7], and lentiviral therapy resulted in T cell lymphoma in a mouse model of X-SCID due to random integration into oncogenes [8]. Therefore, the ideal gene therapy with viral vectors must ensure targeted integration of a therapeutic *HBB* transgene in the endogenous locus. Otherwise, personalized gene-correction therapy with programmable nucleases and non-viral repair templates such as single-stranded oligonucleotides (ssODNs) must be employed as it is less likely to randomly integrate into the genome and result in a safe and precise gene editing [9]. Though several studies targeted *HBB* gene with ZFNs, TALENs, and CRISPR/Cas9, no study has ever compared all the three gene-editing platforms simultaneously. Therefore, in the present study, we compared these three approaches for their target efficiency in parallel. Here, we tested the gene correction efficiency of strategically designed ssODNs as repair templates to target *HBB* gene. This is an important measure while editing the highly proliferating stem cell population to avoid clonal selection and thereby triggering oncogenesis.

## Correspondence/findings

**Fig. 1:**
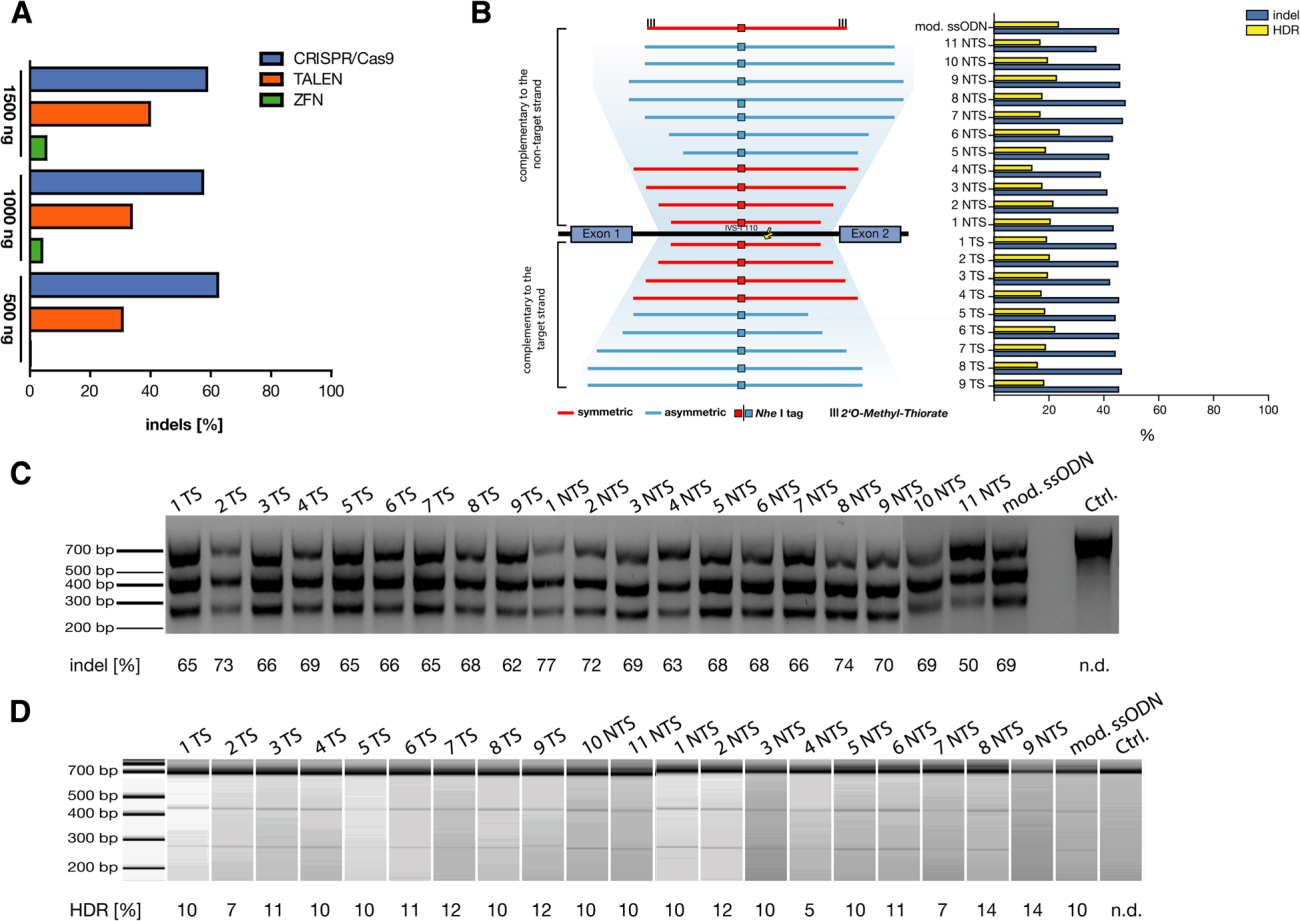
Comparison of three different gene-editing tools at *HBB* promoter and targeting *HBB*^IVS1–110^ locus. **a** HEK-293 cells were transfected with 500, 1000, and 1500 ng of expression plasmid of either ZFNs, TALENs, or CRISPR/Cas9 targeting the promoter of the β-globin gene locus. The indel rate was measured by T7 endonuclease-I (T7EI) assay. Results represent mean values for each concentration, and significant difference was observed among the tools used (*P* < 0.0001). **b** Design of *HBB*^IVS1–110^ sgRNA and ssODN donor templates. K562 cells electroporated with pX330.sg *HBB*^IVS1–110^ plasmid measured for indel rate and HDR. The experiment results from T7EI assay and RFLP assay (**c**, **d**) plotted as a bar graph against utilized ssODNs. **c** The results of T7EI assay analyzed by 1.5% agarose gel electrophoresis. **d** The results of RFLP assay measured in Bioanalyzer using DNA1000 kit (*N* = 3)

First, we designed ZFNs, TALENs, and CRISPR/sgRNA for targeting the promoter region of the *HBB* gene (Additional file 1: Figure S1). The *HBB* gene-targeting efficacy of designed ZFNs, TALENs, or CRISPR/Cas9 was determined by T7 endonuclease-I (T7EI) assay in HEK293 cells. Interestingly, CRISPR/Cas9 exhibited higher indels for all three different concentrations (0.5 μg, 1.0 μg, and 1.5 μg) compared to ZFNs and TALENs (Fig. 1a; Additional file 1: Figure S1). Similar results were observed earlier for the *HBB*^IVS2–654^ mutation where gene-targeting efficiency of CRISPR/Cas9 was superior to TALENs [10]. Therefore, we focused on CRISPR/Cas9 to examine the gene correction efficiency of non-viral repair templates. Several studies have targeted the *HBB* gene using CRISPR/Cas9 system in HSCs, induced pluripotent stem cells, and human embryos [11–16]. Most of these studies were either focused on *HBB* gene addition or targeting sickle cell disease mutation. To the best of our knowledge, this is the first study that attempted to target a common β-thalassemia splicing variant *HBB*^IVS1–110^ (rs35004220), which leads to an alternative splice site and reduced β-globin expression with a non-viral strategy [17].

To target the *HBB*^IVS1–110^ locus, we designed a sgRNA and several ssODNs (Fig. 1b; Additional file 1: Figure S1) with varying lengths of homology arms, symmetrical difference, and chemical modifications and complimentary to the strand targeted or not targeted by the guide-RNA [18]. To evaluate the ability of CRISPR/Cas9 to correct the *HBB*^IVS1–110^ mutation by an exogenous DNA sequence, we introduced an *Nhe*I-tag (restriction site) into the ssODNs that can be stably integrated upon successful homology-directed repair (HDR) (Fig. 1b). We electroporated pX330.sg *HBB*^IVS1–110^ plasmid and ssODN donors harboring *Nhe*I-tag using a Neon system into K562 cells and evaluated the frequency of indels by T7EI assay and HDR-driven *Nhe*I integration by restriction fragment length polymorphism (RFLP) assay (Figs. 1c**/**d). Mean indel frequencies for the targeted loci were 44 ± 18%. We next determined whether any of the rationally designed ssODNs could stimulate gene targeting by HDR and found that most of the tested ssODNs resulted in similar HDR frequencies with the median of 20% (range 5–31%).

**Fig. 2:**
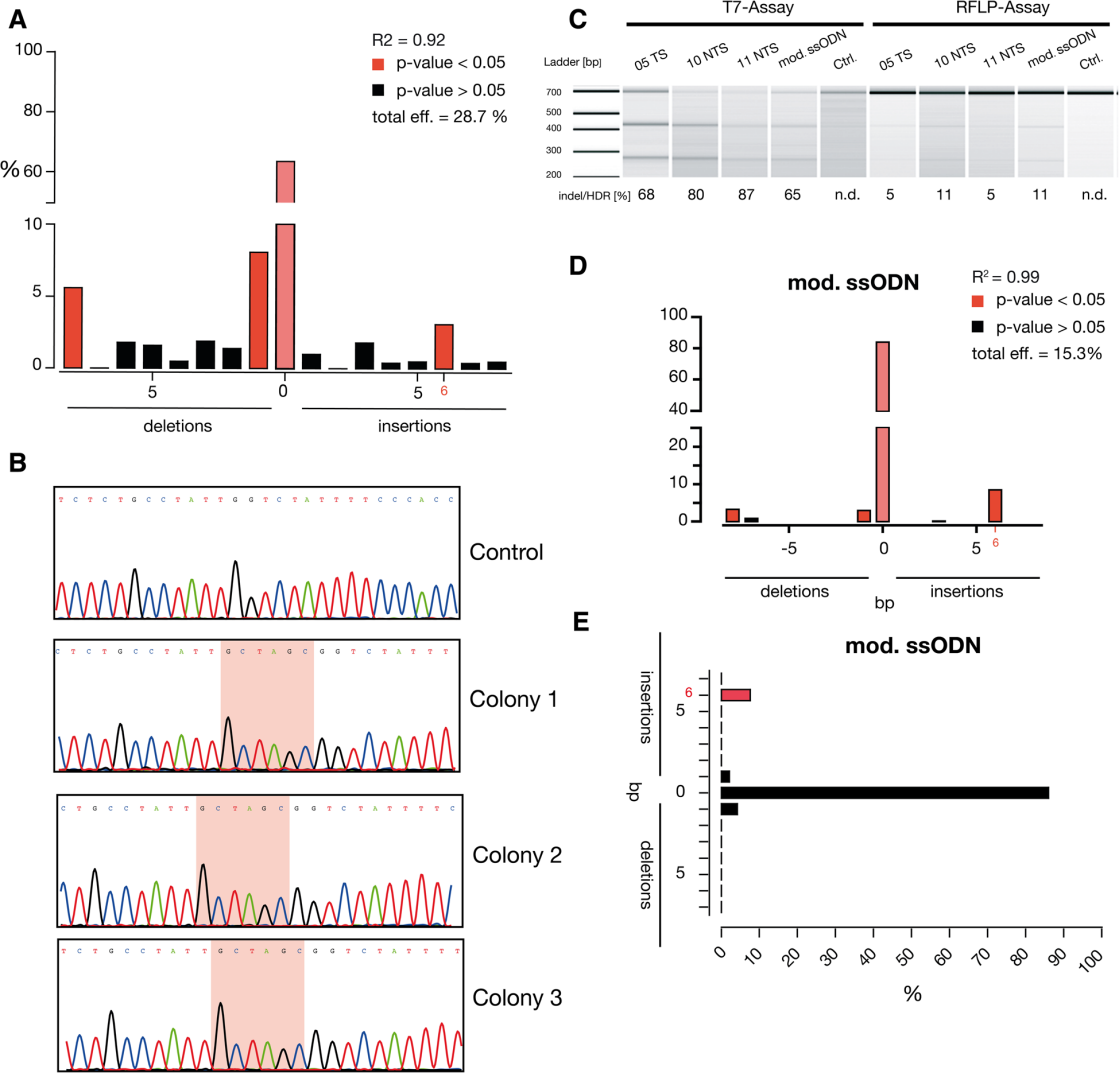
Gene correction of *HBB*^IVS1–110^ in CD34^+^ HSCs using CRISPR/Cas9 and ssODNs. **a** CD34^+^ HSCs co-nucleofected with pX330.sg *HBB*^IVS1–110^ plasmid and 5TS ssODN. Indel frequency was measured by TIDE analysis and 3% of 6 bp insertions were spotted. **b** Single-colony sequencing analysis samples showed successful gene insertion of *Nhe*I restriction-site tag to the *HBB* gene by HDR in pX330.sg *HBB*^IVS1–110^/5TS ssODN-treated sample. **c** Indel and HDR rate of CD34^+^ HSCs co-nucleofected with specified ssODN and Cas9 mRNA measured by T7 assay and RFLP assay in Bioanalyzer using DNA1000 kit. **d** TIDE analysis exhibited the significant enrichment of 6 bp insertions up to 8%. **e** The absolute quantification by next-generation analysis displayed the significant enrichment of 6 bp insertions up to 8% (*N* = 1)

To assess our non-viral correction strategy with bone marrow-derived CD34^+^ HSCs, we co-delivered pX330.sg *HBB*^IVS1–110^ plasmid and several ssODNs. Unlike our results with K562 cells, the indel frequencies in HSCs were relatively low with a median of 30% (range 0–56%) and only one ssODN (5TS) exhibited 3% HDR rate in TIDE analysis (Fig. 2a; Additional file 1: Figure S3.A). We next sought to determine HDR rate for 5TS-ssODN by a semi-quantitative single-colony sequencing and found that 2% (3/172) of clones showed *Nhe*I insertions (Fig. 2b). We observed that pDNA resulted in lower transfection rate and higher cell death in HSCs (data not shown). Due to the low indel rate achieved by pDNA-encoded Cas9, we aimed to increase the efficiency and viability by using mRNA-encoded *Cas*9 as others and ourselves earlier reported the superiority of mRNA over pDNA [19–21]. In addition, transiently expressed *Cas*9 mRNA resulted in reduced off-targets compared to long-term Cas9 expression systems [22]. Therefore, we delivered *Cas*9 mRNA and chemically modified sgRNA with four different ssODNs (chemically modified, 2 PAM-depleted, 5TS) using a 4D-Nucleofector. Genomic analysis with the T7EI assay resulted in high indels ranging from 65% (mod. ssODN) to 87% (11 NTS), and the RFLP assay showed up to 11% integration of *Nhe*I tag at the *HBB*^IVS1–110^ locus (Fig. 2c). Our results clearly imply the superiority of *Cas*9 mRNA over pDNA (Additional file 1: Figure S3.B). We noticed significant enrichment of 6-bp insertion (range 2–9%) by *Nhe*I integration at the target locus for tested ssODNs (Fig. 2d; Additional file 1: Figure S4).

However, the absolute quantification of site-specific insertion events of the *Nhe*I tag at the *HBB*^IVS1–110^ locus by next-Generation sequencing showed 8% correction for modified ssODN (Fig. 2e; Additional file 1: Figure S5). All the analyses spotted improved gene correction for chemically modified ssODN in HSCs as reported earlier [23]. No detectable off-target cleavage was found in six of the in silico predicted off-targets (Additional file 2: Table S4; Additional file 1: Figure S6) for the sgRNA-targeted *HBB*^IVS1–110^ locus**.** We found that CRISPR/Cas9 greatly facilitates targeted genome modification compared to TALENs and ZFNs, and introduction of new DNA sequences in HSCs using *Cas*9 mRNA and ssODN is feasible without viral vectors. However, correction of 8% in HSCs at ex vivo setting is sub-optimal. Therefore, further improvement on HDR efficacy and selection of corrected cells is needed to attain a meaningful gene correction of *HBB* mutations to treat β-thalassemia and other related genetic diseases.

## Online methods

### Design of gene-editing tools

The targeting efficacy at the promoter of the *HBB* gene locus (200 bp upstream of the transcription start site) between three different gene-editing tools (CRSIPR/Cas9, TALENs, and ZFN) was evaluated **(**Additional file 1: Figure S1). The ZFN constructs targeting the promoter were ordered from Sigma-Aldrich (http://www.sigmaaldrich.com). The full amino acid sequences of the ZFN pair are shown in Additional file 2: Data S1. The targeting TALEN pair was designed with the help of the online tool ZiFiT Targeter Version 4.2 (http://zifit.partners.org) and assembled by fast ligation-based automatable solid-phase high-throughput (FLASH). Plasmids encoding TALE repeats harboring different repeat variable di-residues (RVDs) with their FLASH IDs are summarized in Additional file 2: Table S1. The sgRNAs for promoter (5′-AGCCAGTGCCAGAAGAGCCA-3′) and *HBB*^IVS1–110^ (5-GGGTGGGAAAATAGACCAAT-3′) were designed using CRISPR design tool. The respective oligo pairs encoding the 20-nt guide sequences are annealed and ligated into a pX330 vector consisting of a *Sp*Cas9 expression cassette and a U6 promoter driving the expression of chimeric sgRNA. The chemically modified sgRNA for *HBB*^IVS1–110^ mutation was synthesized by incorporating 2-*O*-methyl 3′phosphorothioate (MS) modification at three terminal nucleotides at both the 5′ and 3′ ends.

### Cell culture and transfection

HEK-293 cells were cultured in DMEM (https://www.thermofisher.com) supplemented with 10% FBS and 1% penicillin/streptomycin at 37 °C with 5% CO_2_. In HEK-293 cells, pDNA-encoded gene-editing tools (CRSIPR/Cas9, TALENs, and ZFN) were transfected using Lipofectamin 3000 at three different concentrations of 0.5, 1, and 1.5 μg. K562 cells were cultured in RPMI 1640 (https://www.thermofisher.com) supplemented with 10% FBS and 1% penicillin/streptomycin at 37 °C with 5% CO_2_. In K562 cells, 200 ng of pX330-Chimeric vector targeting *HBB*^IVS1–110^ loci was co-electroporated with 10 pmol of different ssODN using Neon Transfection System (https://www.thermofisher.com). Bone marrow-derived CD34^+^ HSCs from healthy donors were cultured in StemSpan™ serum-free medium II (SFEMII) containing StemSpan™ Cytokine Cocktail 110 (https://www.stemcell.com). 1 × 10^5^ CD34^+^ HSCs were electroporated with 1.2 μg pX330 vector targeting *HBB*^IVS1–110^ mutation and 100 pmol of specified ssODN repair template using Lonza© 4D-Nucleofector™ (Program: EO 100). The similar amount of p.Max eGFP pDNA (1.2 μg) was electroporated as a transfection control. For Cas9 mRNA experiments, 1 × 10^5^ CD34^+^ HSCs were co-electroporated with 5 μg chemically modified sgRNA, 10 μg Ca9 mRNA vector, and 100 pmol of specified ssODN repair template using Lonza© 4D-Nucleofector™ (Program: EO 100).

### Repair templates

The ssODNs were synthesized by Metabion, Germany (www.metabion.com). The ssODNs were designed with an insertion site harboring an *Nhe*I recognition site (GCTAGC). The ssODNs were complimentary to either the strand targeted or not targeted by the gRNA, asymmetric or symmetric to the *Nhe*I tag. The chemically modified ssODN for *HBB*^IVS1–110^ mutation was synthesized by incorporating 2-*O*-methyl 3′phosphorothioate (MS) modification at three terminal nucleotides at both the 5′ and 3′ ends with 72 bp homology arms. The complete details can be found in Additional file 2: Table S2.

### T7 endonuclease assay and RFLP assay

Five days post transfection, genomic DNA was isolated using Merchery-Nagel NucleoSpin Tissue Kit following the manufacturer’s instructions. The promoter region was amplified using the primer pair Prom-For_5′-GTAGACCACCAGCAGCCTAA-3′ and Prom-Rev_5′ TGGAGACGCAGGAAGAGATC-3′, and the region covering *HBB*^IVS1–110^ mutation was amplified using the primer pair IVS1–110-For_ 5′-GGGTTTGAAGTCCAACTCCTAA-3′ and 3′UTR-For_5′-AGAAAACATCAAGCGTCCCATA-3′. The target regions were amplified using the AmpliTag® Gold 360 MasterMix (https://www.thermofisher.com). The cycling parameters for both amplicons were as follows: initial denaturation at 95 °C for 10 min, followed by 40 cycles of denaturation at 95 °C for 30 s, annealing at 55 °C for 30 s, and elongation at 72 °C for 1 min. PCR products were purified by ethanol precipitation, and 1 μg of PCR product was used for T7 endonuclease assay. Likewise, 1 μg of PCR product was used for the RFLP assay where amplicons bearing the *Nhe*I tag upon successful gene correction will result in a specific cleavage after the treatment with the *Nhe*I restriction enzyme. The readout of the T7 endonuclease assay and the RFLP assay were determined on a 1.5% agarose gel and on a Bioanalyzer chip using DNA1000 kit. The band intensities were quantified using ImageJ embedded in Fiji software (www.fiji.sc).

### TIDE analysis and single-colony and next-generation sequencing

For analyzing allele modification frequencies with non-enzymatic assays, we used TIDE (Tracking of Indels by Decomposition) analysis by Sanger-sequencing the purified PCR products used for T7 assay and examined with the online TIDE software (http://tide.nki.nl.) with the respective control sample. To precisely measure the events of site-specific insertion of *Nhe*I tag at *HBB*^IVS1–110^ loci in CD34^+^ HSPCs, we performed two different approaches: (i) Sanger-based single-colony sequencing and (ii) next-generation sequencing. For single-colony sequencing, the *HBB*^IVS1–110^ region was PCR amplified from 5TS ssODN gene-corrected CD34^+^ HSCs and cloned into pJET1.2 vector using CloneJET PCR Cloning kit (https://www.thermofisher.com) and transformed into Top10 competent cells using standard cloning techniques. Closely, 192 (two 96-well plates) single colonies were processed for Sanger sequencing with pJET1.2 forward sequencing primer (5′-CGACTCACTATAGGGAGAGCGGC-3′) and analyzed for the presence for *Nhe*I recognition site (GCTAGC) using Geneious-R6 software. Three of 192 clones resulted in *Nhe*I insertion (1.5%). In case of next-generation sequencing (NGS), new primers were designed with the amplicon length of 150 bp to be sequenced with the Illumina platform (Forward 5′-AGAAACTGGGCATGTGGAGA-3′; Reverse 5′-CCATAACAGCATCAGGAGTGG-3′). Further, barcode-tagged PCR primers were used to multiplex samples (Additional file 2: Table S3), and are adapter ligated, size selected, and bridge amplified and proceeded with amplicon sequencing in HiSeq 4000 system (http://www.illumina.com). The standard R-Package was used for NGS analysis where the sequencing reads were pre-filtered for low-quality reads and mapped to the reference sequence using a BWA tool. Further, the number of indel-carrying sequence reads was calculated using SAM tools. The distribution plot was generated by calculating the size of the indels in sequence and calculating the median percentage for each indel class.

### In vitro transcription (IVT) of Cas9 mRNA

The open reading frame of SpCas9 was PCR amplified from the pX330 vector and sub-cloned into the pVAX.120 vector consisting of a T7 promoter and 120 bp length of a poly-A tail using standard molecular biology techniques. The IVT reaction was performed in linearized plasmid using T7 RNA polymerase in MEGAscript T7 kit (https://www.thermofisher.com). All mRNAs were produced with an anti-reverse CAP analog (ARCA; [m7G(5′)G]) at the 5′ end (https://www.trilinkbiotech.com/). The IVT-mRNAs were made with following chemical modifications in the indicated ratios: 100% Pseudo-UTP and 25% s2-thio-UTP/5-methyl-CTP (https://www.trilinkbiotech.com/). All IVT mRNAs were purified using the MEGAclear kit (https://www.thermofisher.com) and quantified with nano-photometer and bioanalyzed for quality using the RNA6000 kit in Agilent 2100 Bioanalyzer (https://www.agilent.com).

## Statistics

Kruskal-Wallis or Wilcoxon-Mann-Whitney rank-sum tests were applied wherever appropriate to analyze the differences in indel induction among the gene-editing technologies and comparison between encoded pDNA Cas9 and *Cas*9 mRNA using Graphpad Prism v.6.0d (https://www.graphpad.com).

## Additional files

Additional file 1: **Figure S1.** Strategy for targeting the promoter and IVS1–110 mutation of the *HBB* gene. A) The promoter region of *HBB* gene targeted with three different gene-editing tools, *HBB*^IVS1–110^ targeted with CRISPR/Cas9. B) The design of three different gene-editing tools at sequence level. **Figure S2** Comparison of three different gene-editing tools at *HBB* promoter. The complete raw data of Fig. 1a. **Figure S3** Gene correction of *HBB*^IVS1–110^ in CD34^+^ HSCs using pX330.sg *HBB*^IVS1–110^ and ssODNs. A) CD34^+^ HSCs nucleofected with pX330.sg *HBB*^IVS1–110^ plasmid and ssODNs and measured for indel rate by T7 assay and HDR by TIDE analysis. Only 5TS resulted 3% HDR rate in TIDE analysis (as in Fig. 2a). B) Gene-editing capacity of pDNA-encoded Cas9 and mRNA-encoded *Cas*9 were compared, and superiority of Cas9 mRNA was observed (*P* < 0.0001). **Figure S4** TIDE analysis-gene correction of *HBB*^IVS1–110^ in CD34^+^ HSCs using Cas9 mRNA and ssODNs. TIDE analyses of four different ssODNs resulted in varying levels of 6 bp insertions that rely with the ssODN design. Modified ssODN resulted up to 8% HDR rate. **Figure S5** NGS analysis-gene correction of *HBB*^IVS1–110^ in CD34^+^ HSCs using Cas9 mRNA and ssODNs. The absolute quantification of *Nhe*I insertion by NGS analyses for four different ssODNs showed distinct rate of 6 bp insertions and correlate with ssODN design. Modified ssODN resulted up to 8% HDR rate. **Figure S6** Off-target analysis for the in silico predicted sites. The indel rate was measured by T7 endonuclease-I (T7EI) assay for six different off-target sites predicted through in silico (Additional file 2: **Table S4**) in K562 cells. We preselected top three hits in intronic and three hits in an exonic region. (PDF 2219 kb)

Additional file 2 Data S1 The complete amino acid sequences of the *HBB* targeting ZFNs. **Table S1** TALE FLASH IDs and RVDs targeting the promoter of *HBB* gene. **Table S2** Details of ssODNs (sequence, symmetry, and length of homology arms). Table S3 Barcode and sample details of next-generation sequencing. **Table S4** Details of off-target position, primer details, and indel frequency. (PDF 601 kb)
