# Supplementary figures and images for: Gene correction of HBB mutations in CD34+ hematopoietic stem cells using Cas9 mRNA and ssODN donors
Source: Mol Cell Pediatr. 2018 Nov 14;5:9. doi: 10.1186/s40348-018-0086-1 (PMC6236008; doi:10.1186/s40348-018-0086-1)

Figure S1

A

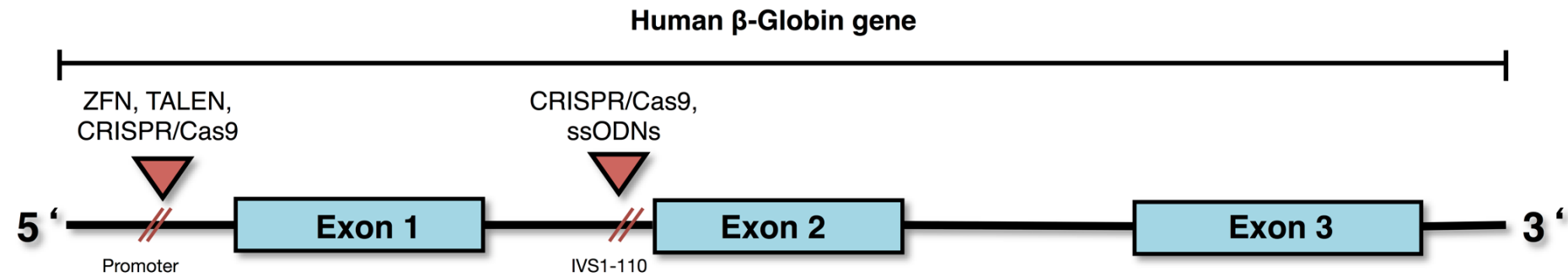

B

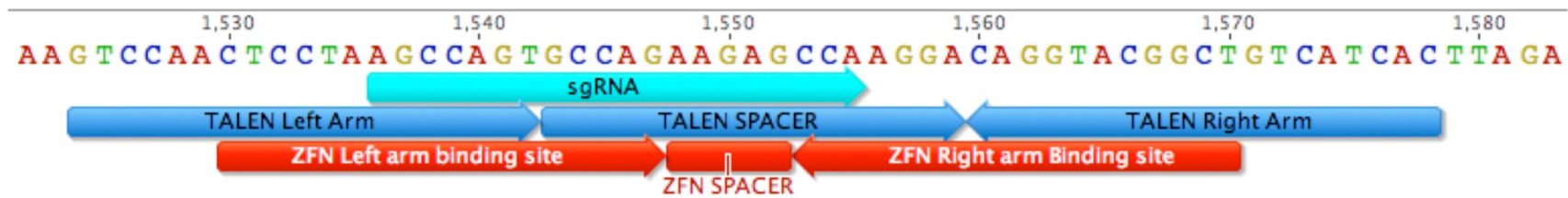

Figure S2

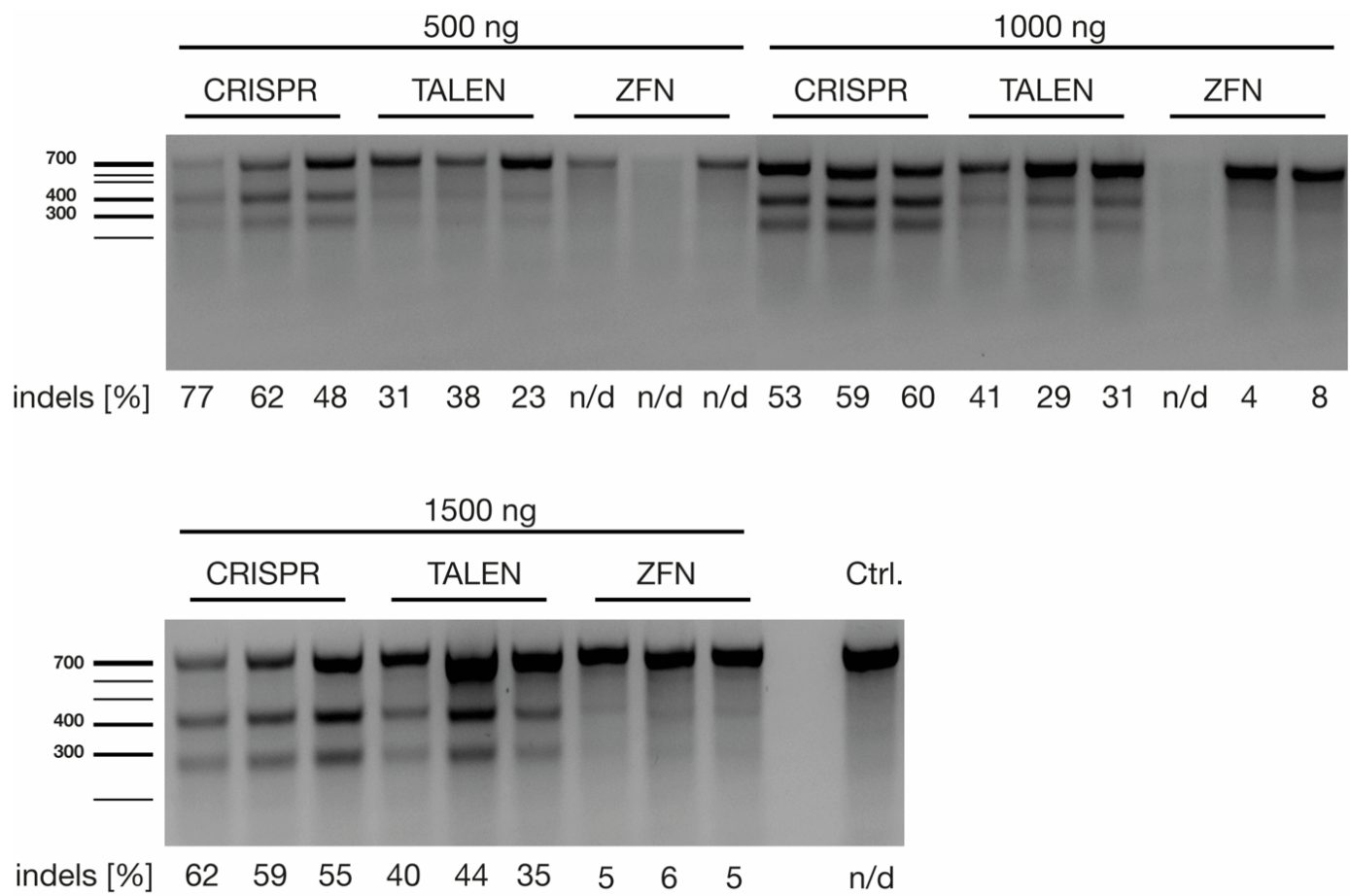

Figure S3

**A**

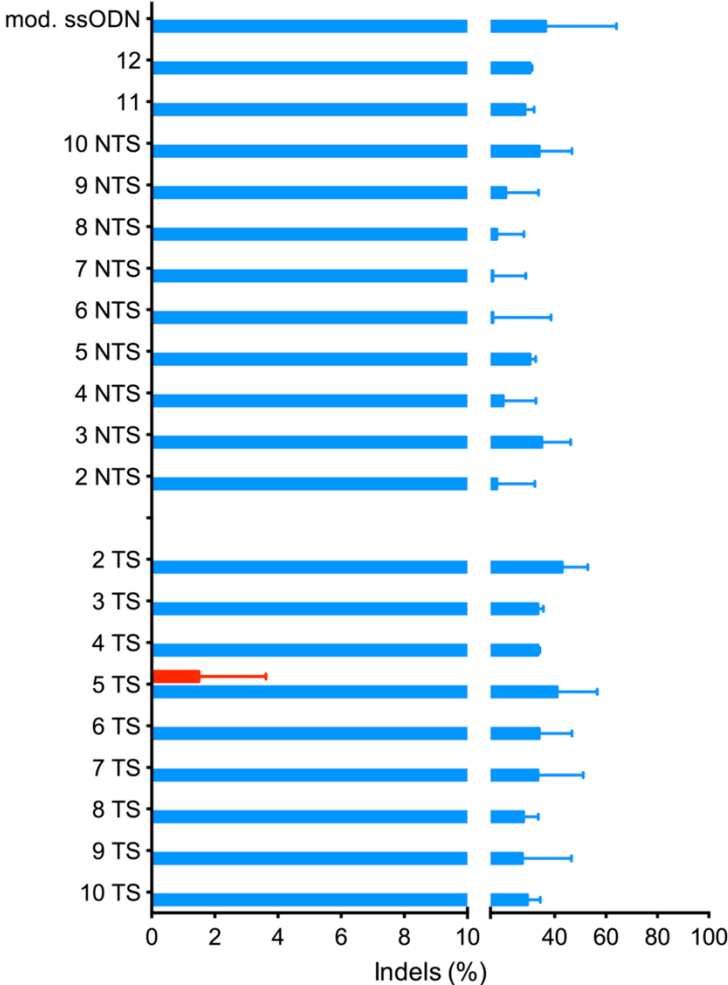

**B**

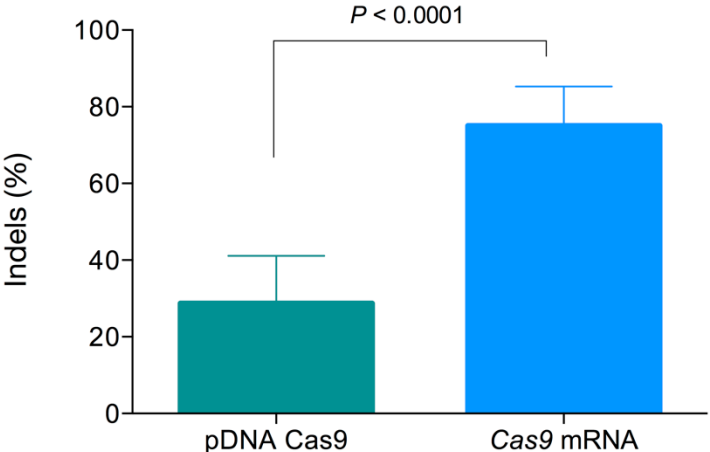

Figure S4

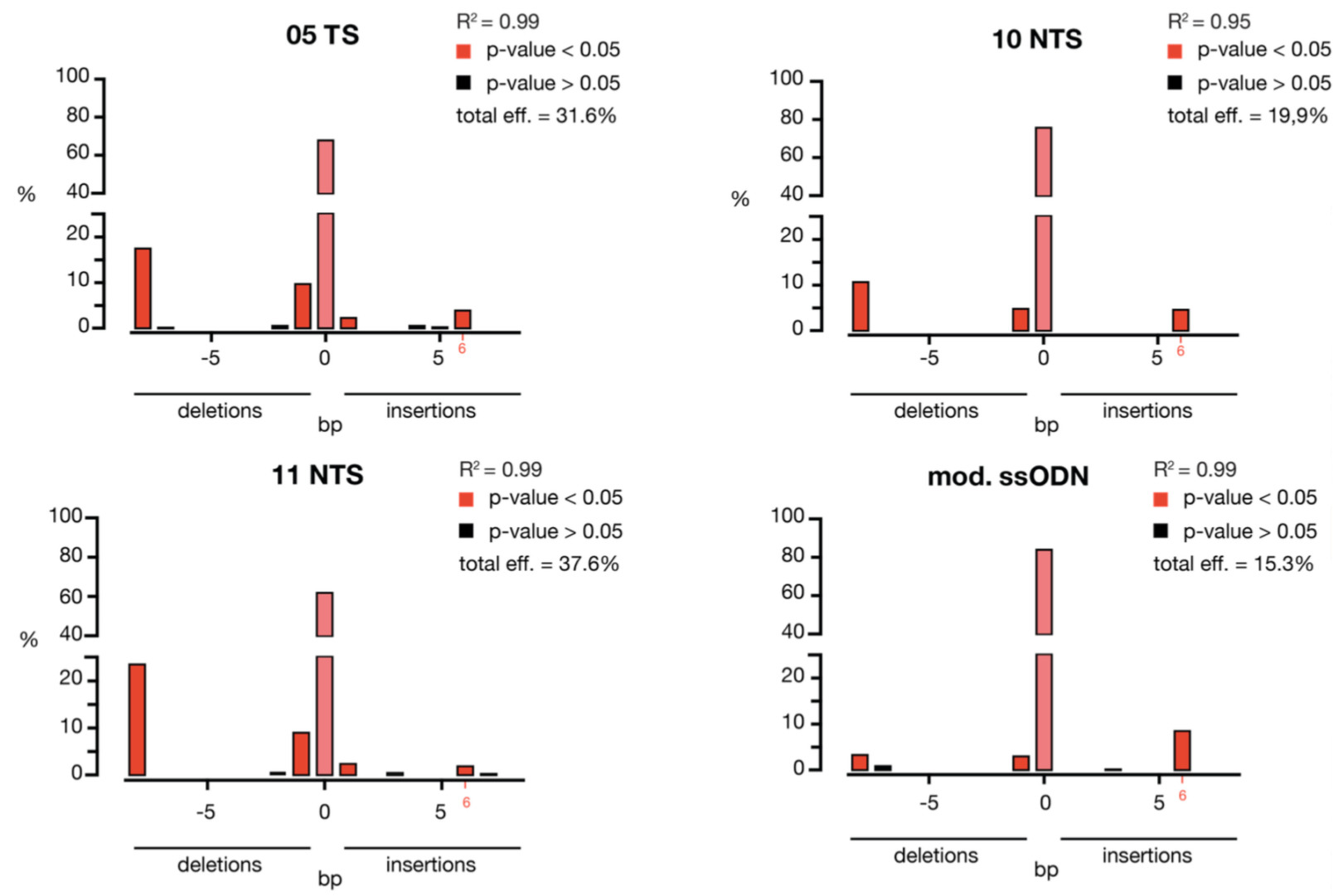

Figure S5

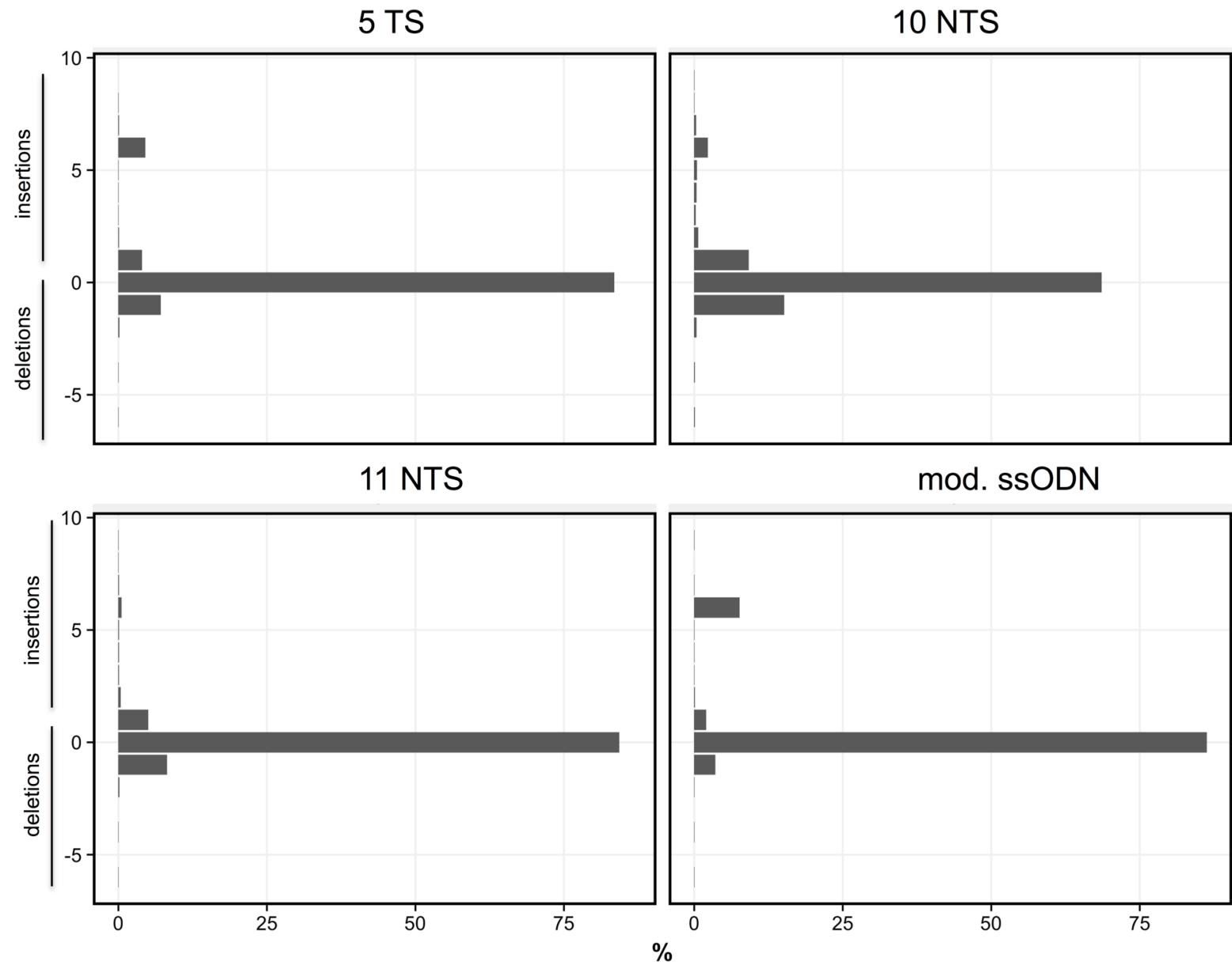

## Figure S6

Supplement: Supplementary file 1 — Figure S1. Strategy for targeting the promoter and IVS1–110 mutation of the HBB gene. A) The promoter region of HBB gene targeted with three different gene-editing tools, HBBIVS1–110 targeted with CRISPR/Cas9. B) The design of three different gene-editing tools at sequence level. Figure S2 Comparison of three different gene-editing tools at HBB promoter. The complete raw data of Fig. 1a. Figure S3 Gene correction of HBBIVS1–110 in CD34+ HSCs using pX330.sg HBBIVS1–110 and ssODNs. A) CD34+ HSCs nucleofected with pX330.sg HBBIVS1–110 plasmid and ssODNs and measured for indel rate by T7 assay and HDR by TIDE analysis. Only 5TS resulted 3% HDR rate in TIDE analysis (as in Fig. 2a). B) Gene-editing capacity of pDNA-encoded Cas9 and mRNA-encoded Cas9 were compared, and superiority of Cas9 mRNA was observed (P < 0.0001). Figure S4 TIDE analysis-gene correction of HBBIVS1–110 in CD34+ HSCs using Cas9 mRNA and ssODNs. TIDE analyses of four different ssODNs resulted in varying levels of 6 bp insertions that rely with the ssODN design. Modified ssODN resulted up to 8% HDR rate. Figure S5 NGS analysis-gene correction of HBBIVS1–110 in CD34+ HSCs using Cas9 mRNA and ssODNs. The absolute quantification of NheI insertion by NGS analyses for four different ssODNs showed distinct rate of 6 bp insertions and correlate with ssODN design. Modified ssODN resulted up to 8% HDR rate. Figure S6 Off-target analysis for the in silico predicted sites. The indel rate was measured by T7 endonuclease-I (T7EI) assay for six different off-target sites predicted through in silico (Additional file 2: Table S4) in K562 cells. We preselected top three hits in intronic and three hits in an exonic region. (PDF 2219 kb) [file 40348_2018_86_MOESM1_ESM.pdf]
