# Supplementary material for: Gene correction of HBB mutations in CD34+ hematopoietic stem cells using Cas9 mRNA and ssODN donors
Source: Mol Cell Pediatr. 2018 Nov 14;5:9. doi: 10.1186/s40348-018-0086-1 (PMC6236008; doi:10.1186/s40348-018-0086-1)
Supplement: Supplementary file 2 — Data S1 The complete amino acid sequences of the HBB targeting ZFNs. Table S1 TALE FLASH IDs and RVDs targeting the promoter of HBB gene. Table S2 Details of ssODNs (sequence, symmetry, and length of homology arms). Table S3 Barcode and sample details of next-generation sequencing. Table S4 Details of off-target position, primer details, and indel frequency. (PDF 601 kb) [file 40348_2018_86_MOESM2_ESM.pdf]

Additional file 2

Data S1 The complete amino acid sequences of the *HBB* targeting ZFNs.

```
>HBB_ZFN Left Arm

   MDYKDHDGDYKDHDIDYKDDDDKMAPKKKRKVGIHGVPAAMAERPFQCRICMRNFSRSDN
   LSAHIRTHTGEKPFACDICGRKFARSDNRTTHTKIHTGSQKPFQCRICMRNFSQSSDLSR
   HIRTHTGEKPFACDICGRKFARRDALLMHTKIHTGSQKPFQCRICMRNFSQSGDLTRHIR
   THTGEKPFACDICGRKFARRDWLPQHTKIHLRGIPAGEERAGGEEVRAAAQAEVRAPRVH
   RADRDRQEQHPGPHPGDEGDGVLHEGVRLQGKAPGRKQKAMKVMEFFMKVYGYRGKHLGG
   SRKPDGAIYTVGSPIDYGVIVDTKAYSGGYNLPIGQADEMERYVEENQTRDKHLNPNEWW
   KVYPSSVTEFKFLFVSGHFKGNYKAQLTRLNHITNCNGAVLSVEELLIGGEMIKAGTLTL
   EEVRRKFNNGEINFRS*

>HBB_ZFN Right Arm

   MDYKDHDGDYKDHDIDYKDDDDKMAPKKKRKVGIHGVPAAMAERPFQCRICMRNFSQSSD
   LSRHIRTHTGEKPFACDICGRKFARATSCTSIPRYTRAAKSPSSVESACVTSVCATTMRN
   FSLRHHLTRHIRTHTGEKPFACDICGRKFAQSGTRKTHTKIHTGSQKPFQCRICMRNFSR
   SDHLSQHIRTHTGEKPFACDICGRKFAQKATRITHTKIHLRGSQLVKSELEEKKSELRHK
   LKYVPHEYIELIEIARNSTQDRILEMKVMEFFMKVYGYRGKHLGGSRKPDGAIYTVGSPI
   DYGVIVDTKAYSGGYNLPIGQADEMQRYVKENQTRNKHINPNEWWKVYPSSVTEFKFLFV
   SGHFKGNYKAQLTRLNRKTNCNGAVLSVEELLIGGEMIKAGTLTLEEVRRKFNNGEINF*
```

Table S1: TALE FLASH IDs and RVDs targeting the promoter of *HBB* gene

**Plasmids Left Arm**	**374**	**53**	**125**	**134**	**325**	**Backbone**
RVDs	HD	NI NG HD NI	HD NG NG NI	NN NI HD HD	NG HD NI	HD
**Plasmids Right Arm**	**373**	**144**	**166**	**6**	**301**	**Backbone**
RVDs	NI	NN NI NG NG	NN NN HD HD	NI NI HD HD	HD NG NI	NN

<u>Table S2:</u> Details of ssODNs (sequence, symmetry and length of homology arms)

Name	Sequence (5' ....*GCTAGC*(*Nhe*I tag)........... 3')	Symmetry	Homology
**1 TS**	AGACTCTTGGGTTTCTGATAGGCACTGACTCTCTCTGCCTATT***GCTAGC***GGTCTATTTTCCCACCCTTAGGCTGCTGGTGGTCTACCCTTGG	symmetric	43 bp + 43 bp
**2 TS**	GAGACAGAGAAGACTCTTGGGTTTCTGATAGGCACTGACTCTCTCTGCCTATT***GCTAGC***GGTCTATTTTCCCACCCTTAGGCTGCTGGTGGTCTACCCTTGGACCCAGAGGT	symmetric	53 bp + 53 bp
**3 TS**	TGGGCATGTGGAGACAGAGAAGACTCTTGGGTTTCTGATAGGCACTGACTCTCTCTGCCTATT***GCTAGC***GGTCTATTTTCCCACCCTTAGGCTGCTGGTGGTCTACCCTTGGACCCAGAGGTTCTTTGAGTC	symmetric	63 bp + 63 bp
**4 TS**	CAATAGAAACTGGGCATGTGGAGACAGAGAAGACTCTTGGGTTTCTGATAGGCACTGACTCTCTCTGCCTATT***GCTAGC***GGTCTATTTTCCCACCCTTAGGCTGCTGGTGGTCTACCCTTGGACCCAGAGGTTCTTTGAGTCCTTTGGGGAT	symmetric	73 bp + 73 bp
**5 TS**	AGACAGGTTTAAGGAGACCAATAGAAACTGGGCATGTGGAGACAGAGAAGACTCTTGGGTTTCTGATAGGCACTGACTCTCTCTGCCTATT***GCTAGC***GGTCTATTTTCCCACCCTTAGGCTGCTGGTGGTCTA	symmetric	91 bp + 36 bp
**6 TS**	ATAGAAACTGGGCATGTGGAGACAGAGAAGACTCTTGGGTTTCTGATAGGCACTGACTCTCTCTGCCTATT***GCTAGC***GGTCTATTTTCCCACCCTTAGGCTGCTGGTGGTCTA	asymmetric	71 bp + 36 bp
**7 TS**	GGTGAGGCCCTGGGCAGGTTGGTATCAAGGTTACAAGACAGGTTTAAGGAGACCAATAGAAACTGGGCATGTGGAGACAGAGAAGACTCTTGGGTTTCTGATAGGCACTGACTCTCTCTGCCTATT***GCTAGC***GGTCTATTTTCCCACCCTTAGGCTGCTGGTGGTCTA	asymmetric	126 bp + 36 bp
**8 TS**	CAGGTTGGTATCAAGGTTACAAGACAGGTTTAAGGAGACCAATAGAAACTGGGCATGTGGAGACAGAGAAGACTCTTGGGTTTCTGATAGGCACTGACTCTCTCTGCCTATT***GCTAGC***GGTCTATTTTCCCACCCTTAGGCTGCTGGTGGTCTACCCTTGGAC	asymmetric	112 bp + 45 bp
**9 TS**	AGACAGGTTTAAGGAGACCAATAGAAACTGGGCATGTGGAGACAGAGAAGACTCTTGGGTTTCTGATAGGCACTGACTCTCTCTGCCTATT*GC**GCTAGC**TTTGGCCGTA*GGTCTATTTTCCCACCCTTAGGCTGCTGGTGGTCTA	asymmetric	93 bp + 46 bp
**1 NTS**	AGACTCTTGGGTTTCTGATAGGCACTGACTCTCTCTGCCTATT***GCTAGC***GGTCTATTTTCCCACCCTTAGGCTGCTGGTGGTCTACCCTTGG	symmetric	43 bp + 43 bp
**2 NTS**	GAGACAGAGAAGACTCTTGGGTTTCTGATAGGCACTGACTCTCTCTGCCTATT***GCTAGC***GGTCTATTTTCCCACCCTTAGGCTGCTGGTGGTCTACCCTTGGACCCAGAGGT	symmetric	53 bp + 53 bp
**3 NTS**	TGGGCATGTGGAGACAGAGAAGACTCTTGGGTTTCTGATAGGCACTGACTCTCTCTGCCTATT***GCTAGC***GGTCTATTTTCCCACCCTTAGGCTGCTGGTGGTCTACCCTTGGACCCAGAGGTTCTTTGAGTC	symmetric	63 bp + 63 bp
**4 NTS**	CAATAGAAACTGGGCATGTGGAGACAGAGAAGACTCTTGGGTTTCTGATAGGCACTGACTCTCTCTGCCTATT***GCTAGC***GGTCTATTTTCCCACCCTTAGGCTGCTGGTGGTCTACCCTTGGACCCAGAGGTTCTTTGAGTCCTTTGGGGAT	symmetric	73 bp + 73 bp
**5 NTS**	AGACAGGTTTAAGGAGACCAATAGAAACTGGGCATGTGGAGACAGAGAAGACTCTTGGGTTTCTGATAGGCACTGACTCTCTCTGCCTATT***GCTAGC***GGTCTATTTTCCCACCCTTAGGCTGCTGGTGGTCTA	symmetric	91 bp + 36 bp
**6 NTS**	ATAGAAACTGGGCATGTGGAGACAGAGAAGACTCTTGGGTTTCTGATAGGCACTGACTCTCTCTGCCTATT***GCTAGC***GGTCTATTTTCCCACCCTTAGGCTGCTGGTGGTCTA	asymmetric	71 bp + 36 bp

**7 NTS**	GGTGAGGCCCTGGGCAGGTTGGTATCAAGGTTACAAGACAGGTTTAAGGAGACCAATAGAAACTGGGCATGTGGAGACAG AGAAGACTCTTGGGTTTCTGATAGGCACTGACTCTCTCTGCCTATT***GCTAGC***GGTCTATTTTCCCACCCTTAGGCTGCTG GTGGTCTA	asymmetric	126 bp + 36 bp
**8 NTS**	CAGGTTGGTATCAAGGTTACAAGACAGGTTTAAGGAGACCAATAGAAACTGGGCATGTGGAGACAGAGAAGACTCTTGGG TTTCTGATAGGCACTGACTCTCTCTGCCTATT***GCTAGC***GGTCTATTTTCCCACCCTTAGGCTGCTGGTGGTCTACCCTTG GAC	asymmetric	112 bp + 45 bp
**9 NTS**	AGACAGGTTTAAGGAGACCAATAGAAACTGGGCATGTGGAGACAGAGAAGACTCTTGGGTTTCTGATAGGCACTGACTCT CTCTGCCTATTGC***GCTAGC***TTTGGCCGTAGGTCTATTTTCCCACCCTTAGGCTGCTGGTGGTCTA	asymmetric	93 bp + 46 bp
**10 NTS**	AGACAGGTTTAAGGAGACCAATAGAAACTGGGCATGTGGAGACAGAGAAGACTCTTGGGTTTCTGATAGGCACTGACTCT CTCTGAGAATT***GCTAGC***GGTCTATTTTCCCACCCTTAGGCTGCTGGTGGTCTA	asymmetric	91 bp + 36 bp PAM-depleted
**11 NTS**	AGACTCTTGGGTTTCTGATAGGCACTGACTCTCTCTAGAAATT***GCTAGC***GGTCTATTTTCCCACCCTTAGGCTGCTGGTG GTCTACCCTTGG	symmetric	43 bp + 43 bp PAM-depleted
**mod. ssODN**	(*T)(*G)(*G)GCATGTGGAGACAGAGAAGACTCTTGGGTTTCTGATAGGCACTGACTCTCTCTGCCTATT***GCTAGC***GG TCTATTTTCCCACCCTTAGGCTGCTGGTGGTCTACCCTTGGACCCAGAGGTTCTTTGA(*G)(*T)(*C)	symmetric	63 bp + 63 bp chemically modified.

Table S3 Barcode and sample details of next-generation sequencing

S. No.	Name	*Nhe*I	Barcode
1.	Control	No	Not pesent
2	5 TS	Yes	ATCACG/ CGTGAT
3	10 NTS	Yes	CTTGTA/ TACAAG
4	11 NTS	Yes	TGACCA/ TGGTCA
5	mod. ssODN	Yes	ACATGT/ ACATGT

>Reference sequence of NGS (167 bp)

AGAAACTGGGCATGTGGAGACAGAGAAGACTCTTGGGTTTCTGATAGGCACTGACTCTCTCTGCCTATT/GGTCTATTTT
CCCACCCTTAGGCTGCTGGTGGTCTACCCTTGGACCCAGAGGTTCTTTGAGTCCTTTGGGGATCTGTCCACTCCTGATGC
TGTTATGG

/ Cut site

Table S4: **Details of off-target position, primer details and Indels frequency.**

Sites	Sequence	Primers (5'....3')	Intron/Exon (gene)	Locus	Indel Frequency (T7E1 Assay)
HBB IVS-I 110	GGGTGGGAAAATAGACCAATAGG	For: GGGTTTGAAGTCCAACTCCTAA<br>Rev: AGAAAACATCAAGCGTCCCATA	Intron	chr11:+5226803	44±18%
OT-1	TAGTGGAAAAATAGACCAATGGG	For: CTACGTCTGCTGAAACCACA<br>Rev: AATTCCAGGGCATCAGGACA	Intron	chr1:+81738787	0%
OT-2	ATGTGGGAAAATGGACCAATCAG	For: AAGCCAAGCAGAAACCACAG<br>Rev: CTGGGCTAATGGGAAGGGAT	Intron	chr3:-6513388	0%
OT-3	AGATGGGAAAATAGACTAATGGG	For: CTCTCACCCTGAACCACACT<br>Rev: AGCAAAGATGAGCCATGCAG	Intron	chr13:-78904261	0%
OT-4	TGGAGGGAAAACAGACCAAGAAG	For: GAGGTCCTTGGAGATTGGCT<br>Rev: AGCCCCTGTCTAATGGTCAC	Exon (NM_024776)	chr15:-77425989	0%
OT-5	GGGAGGGATCAAAGACCAATAAG	For: GGTGGCAAAATGGAGCTGAT<br>Rev: GCCATGCTCAACTACTGCTC	Exon (NM_030581)	chr16:+74976589	0%
OT-6	GAGTGGGAAAACAGACCAGTAGG	For: TTCCTTCTGTCGGGGTCTTC<br>Rev: TCAGAACTCAGGGTGCAGAG	Exon (NM_032878)	chr19:+36503885	0%
